# Deviations in Peripheral Blood Cell Populations are Associated with the Stage of Primary Biliary Cholangitis and Presence of Itching

**DOI:** 10.1007/s00005-018-0515-9

**Published:** 2018-06-27

**Authors:** Halina Cichoż-Lach, Ewelina Grywalska, Agata Michalak, Agnieszka Kowalik, Michał Mielnik, Jacek Roliński

**Affiliations:** 1 Department of Gastroenterology, Medical University of Lublin, Jaczewski 8, 20-954 Lublin, Poland; 2 Department of Clinical Immunology and Immunotherapy, Medical University of Lublin, Lublin, Poland

**Keywords:** Primary biliary cholangitis, Immune dysfunction, Pruritus

## Abstract

To evaluate the role of Th17, Treg cells, activated T CD3^+^ and B CD19^+^ lymphocytes in primary biliary cholangitis (PBC) patients. 40 female patients with PBC and 20 healthy donors were enrolled in this study. The percentages and absolute counts of Th17, Treg, activated T CD3^+^, B CD19^+^, NK, NKT-like lymphocytes were measured by flow cytometry. Our research revealed significantly lower frequencies and absolute counts of CD4^+^CD25^+^FOXP3^+^ Treg cells (*p* < 0.0001), higher percentages and absolute counts of Th17 cells (IL-17A^+^CD3^+^CD4^+^; *p* < 0.0001 and *p* = 0.009, respectively), CD3^−^/CD16^+^CD56^+^ NK cells (*p* < 0.0001 and *p* = 0.039, respectively), CD3^+^/CD16^+^CD56^+^ NKT-like cells (*p* < 0.0001 and *p* = 0.048, respectively). There were also higher percentages and numbers of B CD19^+^ lymphocytes (*p* = 0.002 and *p* = 0.001, respectively) and higher percentages and absolute counts of activated B CD19^+^CD25^+^ cells (*p* = 0.007 and *p* = 0.002, respectively). Moreover, we observed a statistically significant correlation between the presence of itching and particular peripheral blood subpopulations in PBC patients. Absolute counts of both CD4^+^CD3^+^ cells (*p* = 0.0119) and CD3^+^CD25^+^ cells (*p* = 0.0329) were lower in patients with pruritus. A similar dependency was noted in reference to percentages of NKT-like cells (CD3^+^/CD16^+^CD56^+^; *p* = 0.0359) and (CD3^+^) T lymphocytes (*p* = 0.0302). Th17 and Treg cells are involved in the course of PBC. There is also the association between the pruritus and peripheral blood subpopulations.

## Introduction

Primary biliary cholangitis (PBC) is a chronic cholestatic liver disease characterized by progressive destruction of small size intrahepatic bile ducts leading to cholangitis. Among genetic and environmental factors, also the immunological background has conclusively been shown to contribute to the pathogenesis of PBC. Presence of serum anti-mitochondrial antibodies (AMA) constitutes the marker of PBC, noticed in 90–95% of patients (Lindor et al. 2009). PBC is the most frequent autoimmune cholestatic liver disease in humans. Pathological appearance of PBC is inseparably connected with peripheral blood cell subpopulations, particularly T-regulatory- (Treg) and T-helper 17 (Th17) lymphocytes. Nevertheless, their role in PBC pathogenesis remains still uncertain. Scientists proved that manifestation of autoimmunity in humans is inseparably linked to the quantitative and qualitative irregularities of Treg lymphocytes (Chen et al. 2012; Du et al. 2013; Furuhashi et al. 2013; Klatka et al. 2014). It has been shown that activated Treg lymphocytes can inhibit the immunological response irrespective of the antigen (van Herwijnen et al. 2012). Moreover, Th17 lymphocytes are also directly involved in the course of various autoimmune diseases; nevertheless a certain mechanism of their action differs (Murdaca et al. 2011; Pesce et al. 2013; Singh et al. 2013; Vargas-Lowy et al. 2012)..The aim of this research was to describe the percentages and absolute counts of Th17, Treg, as well as activated T CD3^+^ and B CD19^+^ lymphocytes in patients with newly diagnosed PBC and to assess the relationships between analyzed cell subsets and selected clinical parameters (presence of itching and the degree of PBC severity).

## Materials and Methods

### Ethics Statement

The research protocol was approved by the Ethics Committee at the Medical University of Lublin (No. 131/2013) and all patients gave their written informed consent.

### Patients and Controls

Peripheral blood was obtained from 40 previously untreated female patients with PBC (mean age 52.4 ± 12.5; range 37–68 years), and from 20 sex-matched healthy donors (mean age 49.8 ± 6.7; range 36–71 years). The diagnosis of PBC was based on typical diagnostic criteria: (1) biochemical evidence of cholestasis based mainly on elevated alkaline phosphatase activity, (2) presence of AMA and (3) histologic evidence of nonsuppurative destructive cholangitis and destruction of interlobular bile ducts in liver biopsy. All examined patients fulfill all three diagnostic criteria. The degree of severity of PBC was evaluated by histologic stages of PBC. This parameter was described in all patients and they were divided into four groups, according to histologic stages of PBC (I, portal stage—7 patients; II, periportal stage—16 patients; III, septal stage—11 patients; IV, cirrhotic stage—6 patients). Before the initiation of treatment of PBC, lymphocyte subsets, values of peripheral blood cell count parameters, and immunoglobulin serum levels were measured. Itching of the skin was observed in 18 patients. The severity of pruritus was assessed according to visual analog scale (VAS) questionnaire and the mean result was 4.1/10 points. Baseline characteristics of the patients are shown in Table 1. None of the patients and controls had signs of infection at the time of investigation and for a month before collection of the samples and none had been taking drugs with known influence on the immune system, including oral contraceptives. None of the patients or healthy participants had undergone a blood transfusion. Persons with a history of allergic diseases, as well as other autoimmune diseases, were excluded from the study.

**Table 1:** The demographic characteristics and results of laboratory tests in examined patients

Parameter	PBC patients (*n* = 40)	Controls (*n* = 20)	*p* value
Stage of disease	I, 7 pts; II, 16 pts; III, 11 pts; IV, 6 pts		
Presence of pruritus	*n* = 18		
AMA positivity (≥ 1:40)	*n* = 40		
Age (years)	52.4 ± 12.5	49.8 ± 6.7	NS
Gender F/M	34/0	20/0	NS
ALP activity [U/l]	254.2 ± 98.2	98.1 ± 27.2	0.0015*
GGT activity [U/l]	245.2 ± 125.8	39.67 ± 16.5	0.0001*
ALT [U/l]	67.3 ± 28.5	24.0 ± 11.7	0.0325*
AST [U/l]	43.1 ± 17.4	21.8 ± 8.2	NS
Prothrombin time [s]	12.8 ± 1.3	12.1 ± 2.3	NS
Bilirubin [mg/dl]	3.4 ± 1.8	0.8 ± 0.4	0.0032*
IgM [IU/ml]	317.9 ± 115.2	327.3 ± 127.5	NS
IgG [IU/ml]	1345.3 ± 234.7	1543 ± 247.9	NS
Albumin [g/dl]	3.6 ± 1.3	3.9 ± 1.0	NS
PTC [10^9^/l]	197.4 ± 67.3	234.8 ± 98.4	NS

Age and results of laboratory tests are presented as mean ± SD (standard deviation)

*NS* not significant, *n* number of patients, *pts* patients, *AMA* antimitochondrial antibodies, *F* females, *M* males, *ALS* alcoholic liver steatosis, *ALT* alanine aminotransferase, *ASH* alcoholic steatohepatitis, *AST* aspartate transaminase, *ALP* alkaline phosphatase, GGT gamma-glutamyl transferase, *IgM* immunoglobulin M, *IgG* immunoglobulin G, *PTC* platelet count

*Statistically significant differences vs. controls, *p* < 0.05

### Blood Sampling

Venous blood samples (peripheral blood) were collected from the studied patients and controls by venipuncture using sterile, lithium heparin-treated tubes (S-Monovette, SARSTEDT, Aktiengesellschaft & Co., Nubrecht, Germany).

### Isolation of Peripheral Bood Cells and the Detection of Th17 and Treg Cells

Peripheral blood mononuclear cells (PBMCs) were aseptically separated by a standard density gradient centrifugation (Gradisol L, Aqua Medica, Poland).

**Fig. 1:**
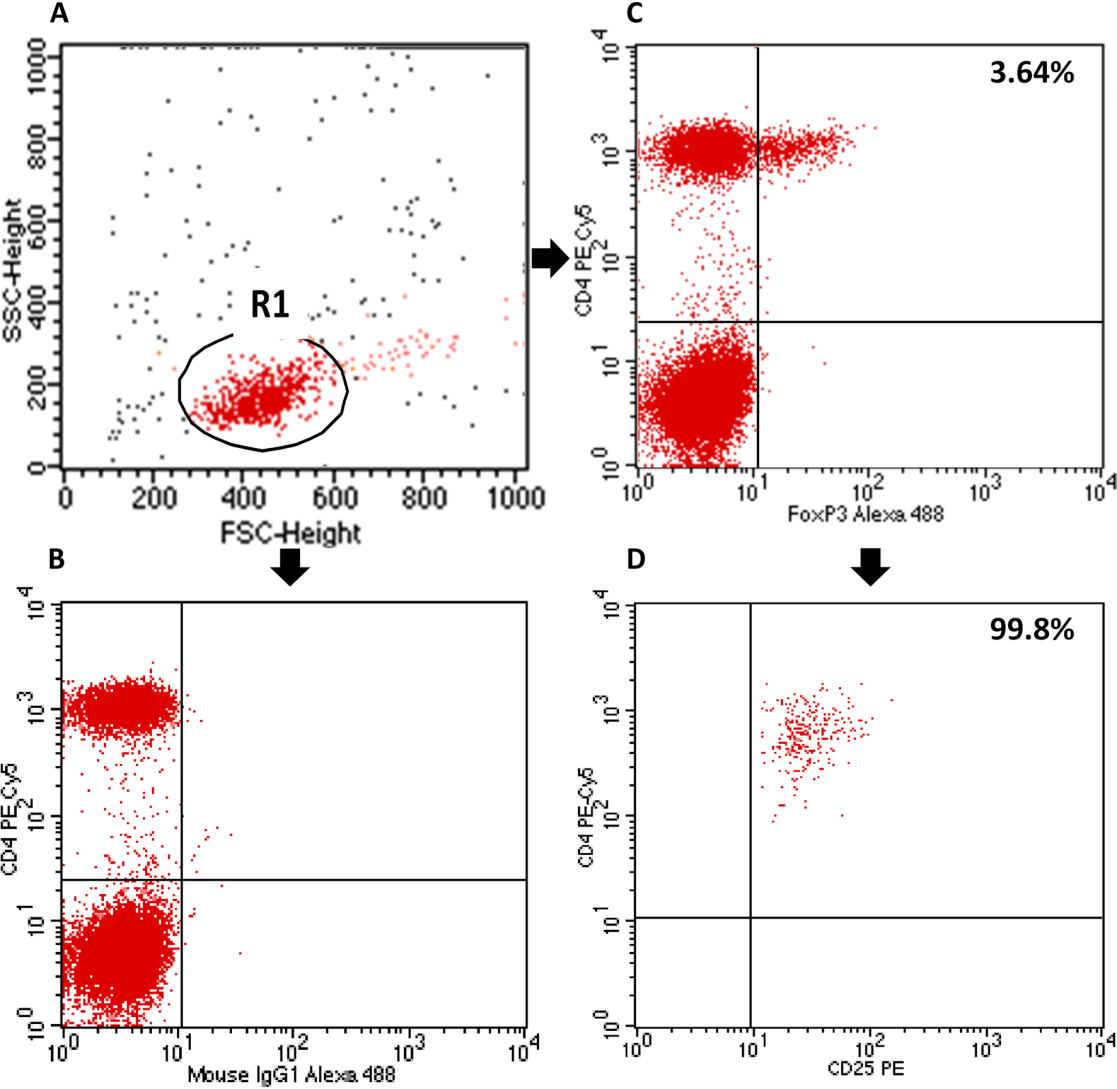
Flow cytometric analysis of T regulatory cells (Treg). First, lymphocytes were gated (R1) on the basis of FSC (forward scatter channel) vs. SSC (side scatter channel) signals. CD4^+^ lymphocytes were then gated and the proportion of CD25^+^FOXP3^+^ cells among the CD4^+^ cells was estimated. **a** Lymphocyte gating. **b** Isotype control. **c** Sample analysis of FOXP3^+^ in CD4^+^ cells (Treg—3.64%). **d** 99.8% of CD4^+^FOXP3^+^ cells co-express CD25 antigen

Tregs in the peripheral blood were assessed by analyzing the expression of the CD4 and CD25 cell surface antigens, as well as the expression of the intracellular FOXP3 antigen using a BD FACSCalibur flow cytometer (BD Biosciences, USA). The percentage of CD4^+^CD25^+^FOXP3^+^ Tregs in the CD4^+^ T lymphocyte subpopulation was determined using the Human Treg Flow kit (FOXP3 Alexa Fluor 488/CD4 PE-Cy5/CD25 PE, BioLegend, USA) according to the manufacturer’s instructions (Fig. 1).

**Fig. 2:**
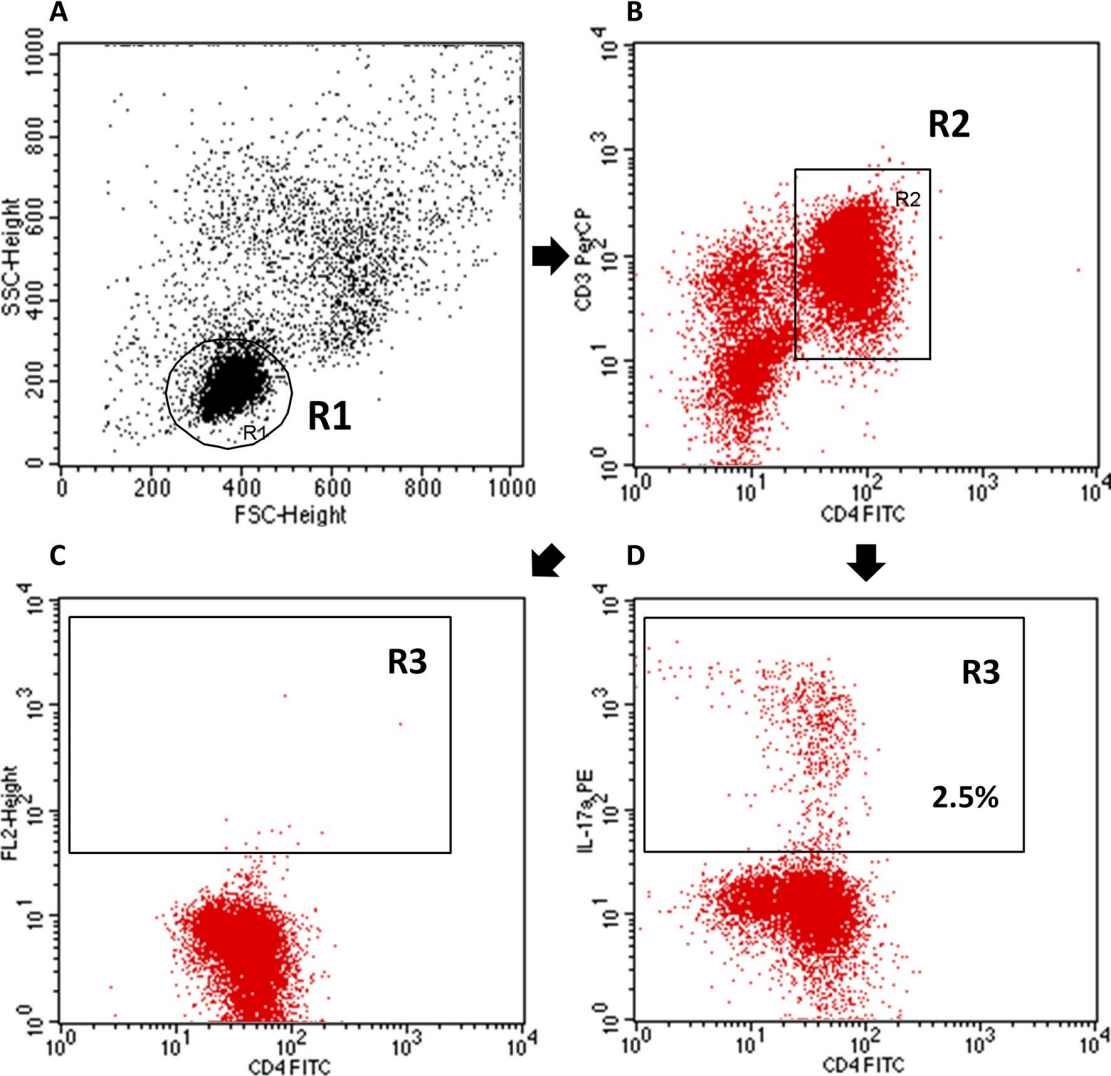
Representative flow cytometric analysis of Th17 cells. First, lymphocytes were gated (R1) on the basis of FSC (forward scatter channel) vs. SSC (side scatter channel) signals. The second step was gating (R2) of CD4^+^ lymphocytes and subsequently estimation of IL-17A^+^ cells among CD4^+^ cells. **a** Lymphocyte gating. **b** Gating of CD3^+^CD4^+^ lymphocytes. **c** Isotype control. **d** Sample analysis of IL-17A^+^ in CD3^+^CD4^+^ cells (R3—2.5%)

For the detection of Th17 cells, PBMCs were resuspended in RPMI-1640 culture medium (Sigma-Aldrich, USA) containing 5% human albumin (Baxter, USA), 2 mM l-glutamine, 100 U/ml penicillin (Sigma, Germany), and 100 µg/ml streptomycin (Sigma-Aldrich, USA). We stimulated mononuclear cells for 5 h at 37 °C in 5% CO_2_ with 25 ng/ml of phorbol 12-myristate 13-acetate- PMA, (Sigma Aldrich, USA) and 1 µg/ml of ionomycin (Sigma-Aldrich, USA) in the presence of 1 µg/ml of Brefeldin A (Sigma-Aldrich, USA), which blocks the intracellular transport processes resulting in the accumulation of cytokine proteins on the Golgi complex. Next, PBMCs were collected, washed with PBS without Ca^2+^ and Mg^2+^ solution and prepared at a final concentration of 10^6^ cells/100 µl per tube. Then, the cells were stained with anti-CD3 PE-Cy5 and anti-CD4 fluorescein-isothiocyanate (FITC)-conjugated monoclonal antibodies (Becton Dickinson, USA). Following the surface staining, cells were fixed and permeabilized using Cytofix/Cytoperm Fixation/Permeabilization Kit (BD Biosciences, USA) according to the manufacturer’s instructions. The permeabilized cells were stained with PE-conjugated anti-human IL-17A monoclonal antibody (MACS Miltenyi Biotec, Germany) or a PE IgG1 isotype control (Fig. 2). Finally, cells were washed and analyzed by flow cytometry, performed on a BD FACSCalibur System (BD Biosciences, USA) (Darmochwał-Kolarz et al. 2012; Hus et al. 2013, 2017; Klatka et al. 2014).

### Immunophenotyping of Natural Killer and Natural Killer T-Like Cells as well as T CD3^+^ and B CD19^+^ Lymphocytes

**Fig. 3:**
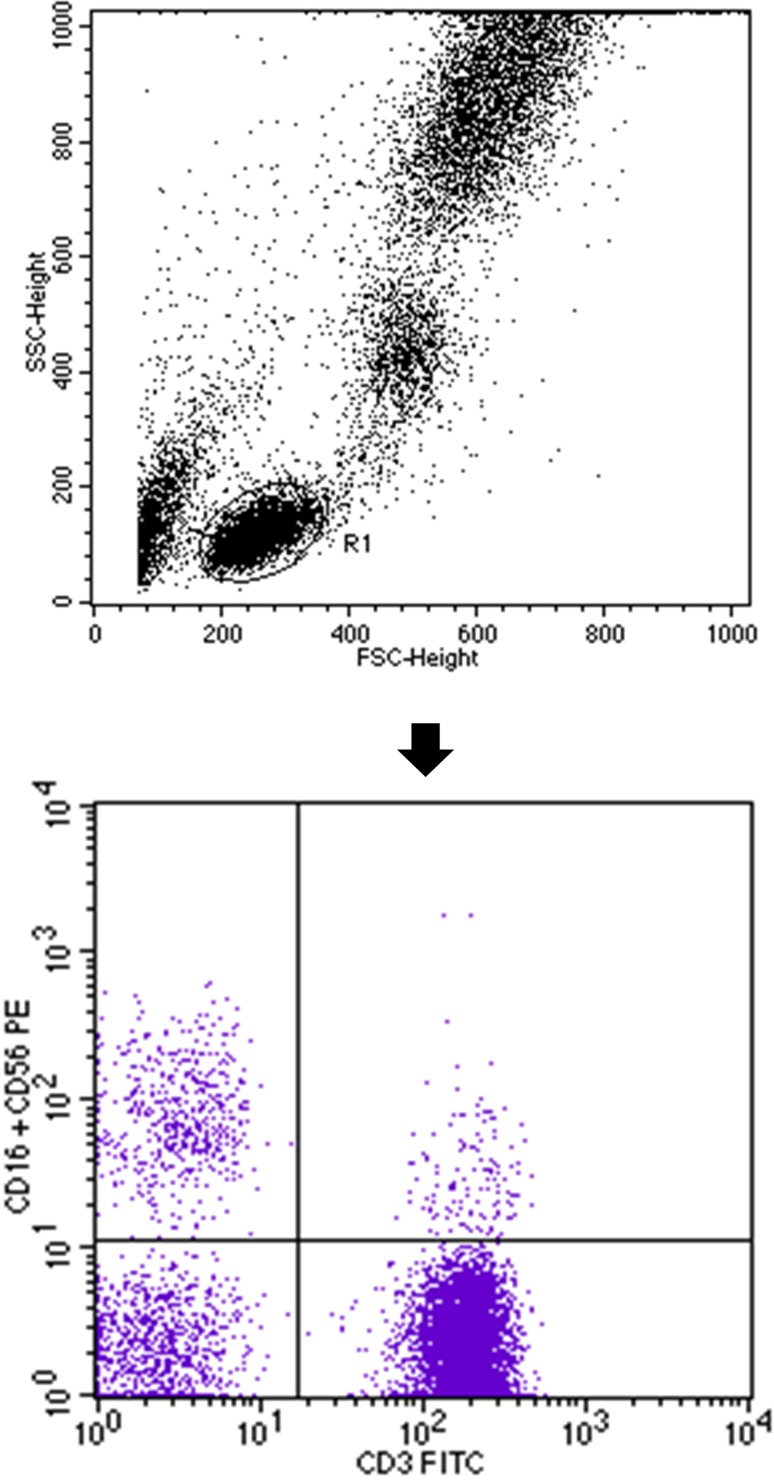
Flow cytometric analysis of NK- and NKT-like cells. First, lymphocytes were gated (R1) on the basis of FSC (forward scatter channel) vs. SSC (side scatter channel) signals (upper dot plot). Finally, dot plot CD3^+^ FITC vs. CD16^+^CD56^+^ PE was established. The upper left quadrant of the bottom dot plot represents the percentage of CD3^−^/CD16^+^CD56^+^ (NK) cells and the upper right quadrant of the bottom dot plot represents the percentage of CD3^+^/CD16^+^CD56^+^ T (NKT-like) cells

Percentages of natural killer (NK) and natural killer T-like (NKT-like) cells were evaluated with flow cytometry using monoclonal antibody anti-CD3 FITC/CD16^+^CD56 PE (BD Biosciences, USA), which allowed for simultaneous assessment of T CD3^+^ lymphocytes and NK cells CD3^−^/CD16^+^CD56^+^. During analysis, the NKT-like CD3^+^/CD16^+^CD56^+^ population was also determined (Fig. 3). A standard, whole-blood assay with erythrocyte cell lysis was used to prepare the peripheral blood samples. Immunofluorescence studies on T CD3^+^ and B CD19^+^ cell subsets were performed using a combination of the following monoclonal antibodies: anti-CD3 FITC/CD19 PE and anti-CD4 FITC/CD8 PE/CD3 PE-Cy5 (BD Biosciences, USA). To determine the activated peripheral blood cells, we used the anti-CD25 PE-Cy5 mouse monoclonal antibody (BD Biosciences, USA). “Cleanness” of lymphocyte gateway was evaluated by examining the distribution of cells in the coordinates of CD45/CD14 (BD Biosciences, USA). The percentage of positive cells was measured from a cut-off set using isotype-matched nonspecific control antibody. Three-colour immunofluorescence analyses were performed using a FACSCalibur flow cytometer (BD Biosciences, USA) equipped with 488 nm argon laser. A minimum of 10,000 events was acquired and analyzed using CellQuest Software (BD Biosciences, USA). The percentage of cells expressing surface markers were analyzed (Hus et al. 2011; Klatka et al. 2017; Nowicka et al. 2017; Pasiarski et al. 2015).

### Statistical Analysis

Statistical analysis of the results was conducted using Statistica 9.0. The demographic date and results of laboratory tests were presented as the mean value ± SD and Student’s t test was used to compare these results. Deviation from normality was evaluated by Kolmogorov–Smirnov test. Data were expressed as the median and range (minimum–maximum). The Mann–Whitney *U* test and Kruskal–Wallis test were used for between-group comparisons because of non-normal distribution. Spearman correlation analyses were used to verify the correlation. All probability values were two-tailed, and a value of *p* less than 0.05 was considered statistically significant.

## Results

The demographic characteristics and results of laboratory tests in examined patients and controls are presented in the Table 1.

### Differences in Tested Parameters between the Study and Control Groups

Our investigation revealed noteworthy differences in the tested parameters between the study and control groups (Table 2). Significantly lower frequencies and absolute counts of CD4^+^CD25^+^FOXP3^+^ Treg cells were found in the study group in comparison to healthy controls (in both cases *p* < 0.0001). Higher percentages and absolute counts of IL-17A^+^CD3^+^CD4^+^ Th17 lymphocytes were found in the peripheral blood of PBC patients than in the control group (*p* < 0.0001 and *p* = 0.009, respectively). Statistically higher percentages and absolute counts of CD3^−^/CD16^+^CD56^+^ NK cells were found in the study group than in the control group (*p* < 0.0001 and *p* = 0.039, respectively). The frequencies and absolute counts of CD3^+^/CD16^+^CD56^+^ NKT-like cells were also significantly higher in PBC patients than in healthy donors (*p* < 0.0001 and *p* = 0.048, respectively). There were no significant differences between the frequencies and absolute counts of T CD3^+^ cells among analyzed groups (in both cases *p* > 0.1). However, higher percentages as well as numbers of activated T CD3^+^CD25^+^ lymphocytes were found in PBC patients comparing to controls (*p* = 0.002 and *p* = 0.001, respectively). Our research revealed also that in the study group there were higher percentages and numbers of B CD19^+^ lymphocytes (*p* = 0.002 and *p* = 0.001, respectively), as well as higher percentages and absolute counts of activated B CD19^+^CD25^+^ cells (*p* = 0.007 and *p* = 0.002, respectively).

**Table 2:** Differences in the tested parameters between the study and control groups

Parameter	PBC patients (*n* = 40)		Controls (*n* = 20)		*Z*	*p* value
	Me	Range, min–max	Me	Range, min–max		
Lymphocytes [× 10^3^/ul]	1.51	0.14–13.40	2.15	1.26–3.12	− 2.723	0.006**
CD4^+^CD25^+^FOXP3^+^ Treg cells [%]	3.79	0.65–7.20	7.91	5.42–11.26	− 5.660	0.000***
CD4^+^CD25^+^FOXP3^+^ Treg cells [× 10^3^/ul]	0.06	0.00–0.16	0.18	0.07–0.33	− 5.123	0.000***
IL-17A^+^CD3^+^CD4^+^ Th17 lymphocytes [%]	13.53	3.54–32.75	5.16	0.34–15.65	4.550	0.000***
IL-17A^+^CD3^+^CD4^+^ Th17 lymphocytes [× 10^3^/ul]	0.24	0.03–1.56	0.12	0.00–0.46	2.615	0.009**
CD3^−^/CD16^+^CD56^+^ NK cells [%]	22.37	10.25–37.50	12.40	5.06–17.18	4.792	0.000***
CD3^−^/CD16^+^CD56^+^ NK cells [10^3^/mm^3^]	0.34	0.02–2.88	0.26	0.11–0.54	2.060	0.039*
CD3^+^/CD16^+^CD56^+^ NKT-like cells [%]	5.75	2.74–10.94	2.79	1.11–4.27	4.792	0.000***
CD3^+^/CD16^+^CD56^+^ NKT-like cells [10^3^/mm^3^]	0.09	0.01–0.74	0.07	0.02–0.13	1.899	0.048*
T lymphocytes CD3^+^ [%]	65.69	45.48–78.78	68.55	13.21–78.63	− 0.909	0.363^NS^
T lymphocytes CD3^+^ [10^3^/mm^3^]	1.66	0.67–3.92	1.65	0.35–2.49	0.556	0.578^NS^
CD3^+^CD25^+^ [%]	11.67	1.60–28.13	7.54	1.08–10.85	3.088	0.002**
CD3^+^CD25^+^ [10^3^/mm^3^]	0.28	0.04–0.91	0.16	0.03–0.38	3.190	0.001**
B lymphocytes CD19^+^ [%]	11.73	7.16–24.53	8.77	5.48–47.40	3.153	0.002**
B lymphocytes CD19^+^ [10^3^/mm^3^]	0.33	0.16–0.94	0.20	0.13–1.26	3.357	0.001***
CD4^+^CD3^+^ [%]	38.93	15.79–49.82	45.0	34.71–48.88	− 3.348	0.0008***
CD4^+^CD3^+^ [10^3^/mm^3^]	0.91	0.31–2.43	1.08	0.57–1.65	− 1.280	0.20006^NS^
CD19^+^CD25^+^ [%]	3.27	0.48–13.06	1.81	0.06–5.12	2.693	0.007**
CD19^+^CD25^+^ [10^3^/mm^3^]	0.08	0.02–0.39	0.04	0.00–0.14	3.135	0.002**

*NS* not significant, *Me* median

^*^p < 0.05; ^**^p < 0.01; ^***^p < 0.001

### The Relationships Between the Tested Parameters and Stages of PBC and Presence of Itching

Our study revealed that the course of PBC might be affected by certain peripheral blood cell subpopulations (Table 3). Current survey demonstrated the correlation between the histologic stage of PBC (II and III) and absolute counts of several peripheral blood subpopulations: CD4^+^CD25^+^FOXP3^+^ Treg cells, (CD3^+^/CD16^+^CD56^+^) NKT-like cells, CD3^+^ T lymphocytes and CD4^+^CD3^+^ cells. A statistically significant higher concentration of Treg cells and NKT-like cells was observed in the III stage PBC patients in comparison to the II stage. Furthermore, a statistically significant lower concentration of CD3^+^ T lymphocytes and CD4^+^CD3^+^ cells was noted in the III stage PBC patients in comparison to the II stage. Additionally, we observed a statistically significant correlation between percentages and absolute counts of CD3^+^CD25^+^ cells in the I and the II histologic stage of PBC; their concentration was higher in the II stage in comparison to the I stage. Only six patients of the research group showed the IV stage of PBC, thus too small number of subjects in this group may explain the lack of statistically significant differences between remained biopsied groups. Interestingly, our investigation found out that there is a statistically significant correlation between the presence of itching and particular peripheral blood subpopulations in PBC patients (Table 4). Absolute counts of both CD3^+^CD4^+^ cells (*p* = 0.0119) and CD3^+^CD25^+^ cells (*p* = 0.0329) were lower in patients with itching. Furthermore, a similar dependency was noted in reference to percentages of NKT-like cells (CD3^+^/CD16^+^CD56^+^; *p* = 0.0359) and (CD3^+^) T lymphocytes (*p* = 0.0302). The remaining examined parameters did not show statistical dependence with the presence of pruritus.

**Table 3:** Differences in absolute counts and percentages of peripheral blood subpopulations according to the stage of PBC

Parameter	Stage of PBC												*p* value
	I: 7 pts			II: 16 pts			III: 11 pts			IV:6 pts			
	Me	Range, min–max	Mean ± SD	Me	Range, min–max	Mean ± SD	Me	Range, min–max	Mean ± SD	Me	Range, min–max	Mean ± SD	
CD4^+^CD25^+^FOXP3^+^ Treg cells [× 10^3^/ul]	0.04	0.02–0.08	0.05 ± 0.02	0.03	0.003–0.16	0.05 ± 0.05	0.09	0.06–0.13	0.10 ± 0.02	0.04	0.04–0.05	0.04 ± 0.01	0.0175*
CD3^+^/CD16^+^CD56^+^ NKT-like cells [10^3^/mm^3^]	0.05	0.03–0.14	0.07 ± 0.04	0.06	0.09–0.18	0.07 ± 0.05	0.15	0.06–0.74	0.19 ± 0.21	0.13	0.12–0.16	0.13 ± 0.03	0.038*
T lymphocytes CD3^+^ [10^3^/mm^3^]	1.66	1.01–2.64	1.86 ± 0.59	1.94	1.21–3.92	2.08 ± 0.69	1.34	0.67–2.46	1.37 ± 0.49	1.66	0.67–3.92	1.56 ± 0.50	0.05*
CD4^+^CD3^+^ [10^3^/mm^3^]	1.03	0.38–1.61	1.09 ± 0.42	1.03	0.66–2.43	1.71 ± 0.44	0.67	0.31–0.90	0.67 ± 0.19	0.91	0.31–2.43	0.10 ± 0.19	0.0086*
CD3^+^/CD25^+^ [%]	8.25	1.60–10.9	7.02 ± 3.25	13.27	4.62–28.13	13.63 ± 6.12	13.02	4.64–14.73	11.45 ± 3.58	10.83	9.11–12.55	10.83 ± 2.43	0.0311**
CD3^+^CD25^+^ [10^3^/mm^3^]	0.18	0.04–0.30	0.18 ± 0.08	0.36	0.14–0.91	0.42 ± 0.24	0.27	0.10–0.60	0.26 ± 0.15	0.26	0.24–0.28	0.26 ± 0.03	0.0161**

*Me* median, *pts* patients, *SD* standard deviation

*Concerns comparison of stages: II and III

**Concerns comparison of stages I and II. Only statistically significant differences are presented

**Table 4:** Differences in absolute counts and percentages of peripheral blood subpopulations with reference to the presence of pruritus

Parameter	PBC patients (*n* = 40)				*p* value
	Pruritus (*n* = 18)		No pruritus (*n* = 22)		
	Me	Range, min–max	Me	Range, min–max	
CD3^+^/CD16^+^CD56^+^ NKT-like cells [%]	4.35	2.74–10.94	6.35	3.74–9.62	0.0359
T lymphocytes CD3^+^ [%]	62.62	52.74–73.94	70.07	45.48–78.78	0.0302
CD4^+^CD3^+^ [10^3^/mm^3^]	0.86	0.31–1.61	1.03	0.38–2.43	0.0119
CD3^+^CD25^+^ [10^3^/mm^3^]	0.25	0.04–0.60	0.31	0.14–0.91	0.0329

Only statistically significant differences are presented

*Me* median, *n* number of patients

## Discussion

Understanding a direct link between T-cell reactivity and autoimmunity in the course of PBC was a great progress in the area of hepatology. PBC-like pathology reflected by inflammatory biliary ductulitis and the presence of AMA in serum was presented to accompany T cell abnormalities in genetically based mouse models (Mackay 2007). Our study supports this idea since we found the Treg deficiency as well as elevated CD25^+^ cells in the peripheral blood of PBC patients in comparison to healthy controls. Treg cells play a pivotal role in self-tolerance and controlling excessive immune responses. Their reduced number in PBC is a well-known phenomenon. It could be responsible for the loss of immune tolerance and development of autoimmune process. According to the previous studies, aberrations of CD8^+^ cells in PBC patients were also observed. Furthermore, B-cell-activating factor (BAFF) is involved in disorders in PBC affecting Treg cells. It is a crucial survival factor in the course of B-cell maturation. The level of BAFF in patients with diagnosed PBC is elevated. It has been confirmed that activated B cells are able to inhibit Treg cell proliferation. Interestingly, BAFF receptors on Treg cells have not been found. Investigations performed on PBC patients revealed that BAFF is responsible for the Treg cell apoptosis and inhibited cytokine production by activating B cells. These data are especially noteworthy because they suggest the inhibition of BAFF activation as a potential treatment strategy for PBC. What is more, mice with T-cell-restricted expression of a dominant negative form of the transforming growth factor (TGF)-β receptor type II were observed to develop spontaneously autoimmune cholangitis resembling human PBC, with the presence of AMA together with portal lymphocytic infiltration (CD4^+^, CD8^+^). By means of this fact, the deprivation of TGF-β signaling restricted to T cells might be the reason of pathological appearance of PBC (Bernuzzi et al. 2010; Fenoglio et al. 2012; Huang et al. 2014; Wang et al. 2015; Zhang et al. 2013; Yang et al. 2008). Lan et al. (2009) detected elevated number of interleukin (IL)-17 producing cells in liver tissues of PBC patients in comparison to healthy controls. Those authors reported also the presence of IL-17^+^ cells in livers of IL-2-knockout mice (since IL-2 inhibits Th17 cells production) (Lan et al. 2009). Herein, we report that higher levels of Th17 cells are found in the peripheral blood of PBC patients in comparison to healthy controls. Obtained results suggest also that severe immune system deregulation, reflected by high numbers of Th17 cells with additional low frequencies of Treg lymphocytes, may influence the severity of inflammatory response and the progression of disease to such an extent that these parameters correlate with histological stage of PBC. Th17 cells are a key factor in the pathogenesis and the development of many autoimmune disorders, e.g., autoimmune encephalitis, rheumatoid arthritis and inflammatory bowel disease (Brand 2009; Hamburg et al. 2011; Hot and Miossec 2011; Jadidi-Niaragh and Mirshafiey 2011; Noubade et al. 2011). The subpopulation of Th17 cells in the course of PBC is of crucial importance because of IL-17 production. This cytokine was proved to participate in tissue destruction and proinflammatory mediator induction. It is also responsible for the progression of fibrosis in various conditions, like myocardial fibrosis or bleomycin-induced idiopathic pulmonary fibrosis (Feng et al. 2009; Gasse et al. 2011; Wilson et al. 2010). The research conducted on IL-17A-knockout mice with the model of PBC, revealed the reduction of AMA. Scientists showed also that the depletion of Th17 cell cytokines (IL-17A, IL-22) lowers the degree of biliary damage (Kawata et al. 2013). Moreover, IL-17 has a profibrotic profile and is implicated in organ fibrosis. Of note, biliary epithelial cells are able to release Th17-inducible cytokines. Other researchers found Th17 cells to accumulate in the area of damaged bile ducts in PBC patients. The loss of intrahepatic biliary ducts in PBC might be the result of epithelial to mesenchymal transition (EMT). This process stands for the phenotypic reprogram, which results in the loss of epithelial character and acquisition of feature close to fibroblasts. EMT was also shown to be a very early process in the course of PBC. Aforementioned profibrotic characteristics of IL-17 might be responsible for the liver fibrosis in the course of PBC. It may be also a potential target as a management for autoimmune liver diseases (Harada et al. 2009; Huang et al. 2016; Romagnani et al. 2009; Rong et al. 2009; Shi et al. 2015). Subsequently, EMT was also proved to attenuate fibrosis in various pathological states. Investigations revealed that IL-17 is able to activate EMT of alveolar and bronchial epithelial cells. The importance of IL-17 in disease progression requires further investigation, such as studies involving the blocking of IL-17 signals. The exact mechanism of the exuberant Th17 induction in PBC is not clear (Ji et al. 2013; Mi et al. 2011; Vittal et al. 2013). The dysregulation of NK cell induction seems to be another important underlying defect. Data in literature concerning the role of NK cells in the course of cholangitis are scanty and this area should be undoubtedly explored. Our investigation revealed increased frequency of both CD3^−^/CD16^+^CD56^+^ NK cells and CD3^+^/CD16^+^CD56^+^ NKT-like cells in patients with PBC. The destruction of biliary cells in PBC is reported to consist of three main elements: macrophages, biliary epithelial apotopes and AMA. A final cascade leads to the release of proinflammatory cytokines. Furthermore, NK cell cytotoxicity is also widely emphasized because of their ability to synthesize cytokines rapidly. In consequence, they have an impact on quality and quantity of acquired immune responses. Scientists studied a murine model of human PBC with the depletion of NK and NK T cells and observed the suppression of AMA and cytokine release by autoreactive T cells. Interestingly, no statistically important change of portal inflammation was noted (Feuerer et al. 2009; Lanier 2008; Shimoda et al. 2012). According to conducted investigations, NK cells were reported to participate in the destruction of cholangiocytes. Moreover, NK T cells were found to be involved in the exacerbation of PBC (Chuang et al. 2008; Kita et al. 2002; Wu et al. 2011). By means of these data, it becomes quite obvious that innate immune effector mechanisms are inseparably connected with acquired immunity and play a crucial role in the pathogenesis of PBC. Nevertheless, the underlying mechanisms between innate immune effector actions and the development of pathogenically acquired immune responses in PBC remain unknown and still require further research (Berg 2011; Selmi et al. 2009). The limitation of current investigation is relatively a small number of participants. The further study should include the level of IL-17 as one of assessed the parameters in PBC patients. Furthermore, our investigation seems to be the first that demonstrated the correlation between pruritus and concentrations of certain peripheral blood subpopulations in PBC patients. This area should be undoubtedly explored in the future. Results achieved in this survey correlate with data in the literature and suggest immunological background as the key factor leading to PBC and one of the possible targets of treatment.

In conclusion, presented study demonstrates that both Th17 and Treg cells might play an important role in the pathogenesis and treatment of PBC. Reduced number of Treg cells and high level of Th17 cells in PBC could be responsible for the loss of immune tolerance, development of inflammatory, autoimmune process and liver fibrosis in PBC. The progression of the disease might be the result of Th17 cell activation and the release of IL-17. Treg cells and NKT-like cells correlate with histological stages of PBC. There is also the association between the presence of itching and concentrations of particular peripheral blood subpopulations. Undoubtedly, our results lay a solid base for further studies that we would like to continue in the near future. To our knowledge, this investigation is the first one performed in Polish population that assesses the correlation between PBC course and peripheral blood subpopulations.
